# In memoriam: Prof. Dr. Med. Martin Schumacher (1944–2021)

**DOI:** 10.1007/s00234-021-02883-9

**Published:** 2021-12-22

**Authors:** Ansgar Berlis

**Affiliations:** Universitätsklinikum Augsburg A.ö.R., Stenglinstrasse 2, 86156 Augsburg, Germany

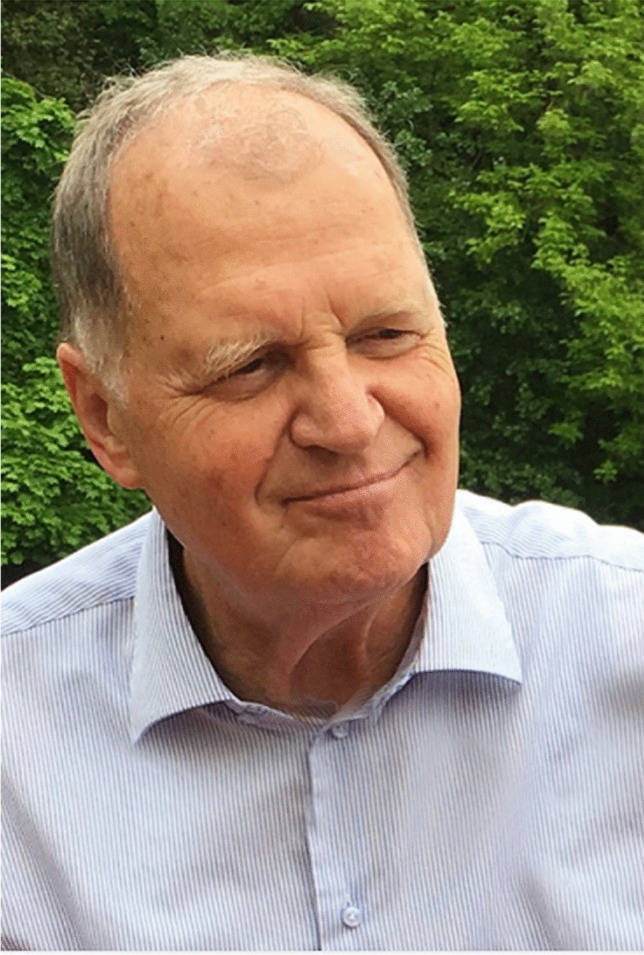



On November 18, 2021, Prof. Dr. Martin Schumacher, former Head of Neuroradiology at the University Hospitals Freiburg, Germany, passed away at the age of 77. Martin Schumacher studied medicine in Freiburg from 1965 to 1971 and received his medical degree after a 1-year research sabbatical in the USA in 1970. Thereafter, he joined the Neurology and Neurosurgery Residency program at the University Hospitals Freiburg and Tübingen. In 1977, Martin moved to Tübingen and received his postdoctoral lecture qualification with the thesis, “Neuroradiology of experimental brain tumors,” in 1980. Following this achievement, he became Head of Neuroradiology and later of Neurology as well at the Christophsbad Hospital in Göppingen, Germany. In 1984, Martin accepted the offer to become the Head of Neuroradiology at the University, where he remained, in spite of calls from the universities of Marburg and Greifswald. Martin Schumacher was supposed to retire in 2011; however, external circumstances meant he assumed responsibility for another 2 years. Prof. Dr. med. Horst Urbach took over the Neuroradiological Department in excellent condition on May 1, 2013. Even so, Martin remained closely connected to Neuroradiology nationally and internationally until his death in November.

Martin Schumacher, Board Certified in Neurology and Neuroradiology, regarded clinical service, diagnostic approaches, and therapeutic choices to be a holistic unit. As one of the German pioneers in Interventional Neuroradiology, he always approached interventional clinic practice with the classical neurosciences’ triad of Neurology, Neurosurgery, and Psychiatry in mind.

As head of Neuroradiology in Freiburg, Martin built up an international network. He visited Jean-Jacques Merland in Paris, Joseph A. Horton in Pittsburgh, Alejandro Berenstein in New York, Charles Kerber in San Diego, and Fernando Vinuela in Los Angeles. In 1986, he began to integrate intra-arterial fibrinolysis in daily practice and used endovascular mechanical devices for the first time at the end of the 1990s. In 1992, Martin was the first neuroradiologist in Germany and Europe to treat a brain aneurysm with the new Guglielmi detachable coils.

Martin’s enthusiasm for neurointerventional surgery inspired his research and teaching in national and international neurointerventional courses, and he knew how to inspire colleagues as a personal teacher and scientist.

Additionally, Martin Schumacher was exceptionally committed to the German and European Societies of Neuroradiology. He served as President of the German Society (DGNR) for two terms, President of the European Society of Neuroradiology (ESNR) from 2006 to 2008, and was an active member of the ESNR Executive Committee from 2000 to 2010. At the 2021 annual ESNR meeting in Geneva, the ESNR Founders Award, Interventional Neuroradiology 2021, was awarded in honor of Martin Schumacher.

Importantly, Martin was responsible for founding the subdivision “Neuroradiology” within the European Union of Medical Specialists (UEMS) and for initiating the European Exchange Program to promote international exchange between young neuroradiologists.

In recognition of his outstanding contributions, Martin Schumacher was awarded Honorary Memberships of the DGNR in 2013, ESNR in 2014, and numerous other national Neuroradiology societies.

Wilhelm Radü, the former Head of Neuroradiology in Basel, Switzerland, recognized Martin Schumacher’s achievements on his 60th birthday: “Only those who cross borders can succeed, and Martin Schumacher is the man who understood and understands overcoming geographically and neuroradiological boundaries.”

Together with his wife Grit, his two sons with partners and four grandchildren, many friends will miss him and cherish his inspiring memory.

